# A Farewell to Arms

**DOI:** 10.1007/s12471-016-0915-6

**Published:** 2016-11-09

**Authors:** E. E. van der Wall

**Affiliations:** Netherlands Society of Cardiology/Holland Heart House, Utrecht, The Netherlands

*The great fallacy is the wisdom of old men. They do not grow wise. They grow careful.*

A Farewell to Arms, Ernest Hemingway (1899–1961) [1]

At a certain moment in life everyone has to make important decisions with direct implications for their future. This time has now arrived and I would like to announce that, as of 1 January 2017, I will be stepping down as Editor-in-Chief of the Netherlands Heart Journal (NHJ). Formally, the appointment of an Editor-in-Chief is a matter for both the Publisher and the Netherlands Society of Cardiology (NVVC). Together they arrive at the decision on who is the most appropriate candidate for this position at this moment.

At the 2016 autumn meeting of the NVVC it was officially announced that from 1 January 2017 Professor Jan J. Piek (AMC, Amsterdam) will take over the position of Editor-in-Chief of NHJ, for a well-defined period of time. He will be assisted by local deputy editors. Further details will be given in the NHJ Editor’s Comment of January 2017.

It was 15 years ago, on 1 April 2001, that the Netherlands Heart Journal was founded. It was the offspring of the journal entitled ‘Nederlands Tijdschift voor Cardiologie’ (founded in 1987) and its successor ‘Cardiologie’ (founded in 1994), both journals predominantly publishing in the Dutch language. However, at the turn of this century it was thought wise and timely, by both the Chief Editors and the Publisher, to completely change to the English language and to mainly focus on original and review articles. At that time, the first Editorial Comment in April 2001 ended with the phrase ‘*We hope that you really appreciate our efforts to make the Netherlands Heart Journal a new platform for cardiovascular research in a broadest sense of the word*’! [2]. Our aims at that time were 1) to reach PubMed to become internationally recognised, and 2) to be listed by the Web of Science (WOS), including the Science Citation Index (SCI), in order to obtain an impact factor [3, 4].

Let us briefly go over the main events and achievements of NHJ since 2001. On 1 January 2005, our journal changed its publishing company from Mediselect BV to Bohn, Stafleu & van Loghum (BSL, Houten, the Netherlands), which over time became a division of the Springer Verlag Concern, Germany.

In 2006, NHJ was adopted by PubMed Central (www.pubmedcentral.gov) [4, 5], which is a free full-text archive of biomedical and life sciences journal literature at the US National Institutes of Health’s National Library of Medicine (NIH/NLM). In 2007, NHJ was internationally recognised by PubMed, the most important literature database comprising more than 26 million citations for biomedical literature from MEDLINE, life science journals, and online books (www.pubmed.gov). PubMed has now retroactively listed all NHJ articles (almost 2000!) from its very start in April 2001. In 2008, NHJ was indexed by WOS and received its first impact factor in 2009, which was 1.392 at that time and by 2015 it had increased to 2.062 [6–11].

These major achievements were not given to us for free. It has cost much effort and many years for NHJ to enter the international arena. Once getting there, we have had to battle to stay there and it requires an ongoing fighting spirit to preserve and, more importantly, to improve the scientific level of our journal. This achievement has been made possible, and continues to be possible, through the cooperation and support of many institutions and individuals. In particular the Publisher (predominantly represented by Lieda Meester) together with my former Chief Editors, Professors de Boer, Doevendans, Wilde and Zijlstra, laid the basis for these martial accomplishments. Furthermore, over the years we have received support from the NVVC [12, 13], the Cardiovascular Institute for Education (CVOI) [14], the Interuniversity Cardiology Institute of the Netherlands (ICIN) [15], and the Netherlands Heart Foundation (NHS). Lastly, the true scientific level of a journal is determined by the members of the Editorial Board, in particular the Deputy and the Associate Editors, and of course the reviewers. I fully acknowledge their support for NHJ and for me over the years. In my turn, I sincerely hope I served you well during this 30-year period from 1987–2017.

‘*Great is the art of beginning, but greater is the art of ending*’ the American poet Henry Wadsworth Longfellow (1807–1882) once said. A new editorial period will begin and I am fully confident that NHJ will continue to thrive. In the style of Ernest Hemingway, I say farewell to arms with one of his quotes; ‘*it is now hard to leave the country but it is in no way impossible*’ [1].

## References

[1] Hemingway E (1929). A farewell to arms.

[2] van der Wall EE (2001). Netherlands heart journal: just a change of name or more?. Neth Heart J.

[3] Opthof T, Coronel R (2002). The impact factor of leading cardiovascular journals: where is your paper best cited?. Neth Heart J.

[4] de Boer MJ, van der Wall EE (2008). Towards better cardiovascular journals. Neth Heart J.

[5] van der Wall EE, de Boer MJ, Doevendans PA, Wilde AA, Zijlstra F (2006). Netherlands Heart Journal: accepted into PubMed Central!. Neth Heart J.

[6] van der Wall EE (2010). Increasing recognition of NHJ: a first-time impact factor of 1.4!. Neth Heart J.

[7] van der Wall EE, de Boer MJ, Doevendans PA, Wilde AA, Zijlstra F (2011). Journal metrics for the Netherlands Heart Journal. Neth Heart J.

[8] van der Wall EE (2013). Impact factor 2012 for cardiovascular journals: true impact?. Neth Heart J.

[9] Opthof T (2013). The impact factor of the Netherlands Heart Journal in 2013. Neth Heart J.

[10] Opthof T (2014). Impact factor 2013 of the Netherlands Heart Journal surpasses 2.0. Neth Heart J.

[11] van der Wall EE (2016). Impact factors 2015; NHJ on the rise!. Neth Heart J.

[12] de Boer MJ (2009). The NVVC: here, there and everywhere. Neth Heart J.

[13] Umans VA, van der Wall EE (2014). 80 years of Netherlands Society of Cardiology. Neth Heart J.

[14] Michels HR (2001). Continuing medical education in Europe: NVVC, CVOI, ESC, UEMS and EBAC. Neth Heart J.

[15] van Gilst WH, van der Wall EE (2010). Einthoven dissertation prize 2010. Neth Heart J.

